# Chromatographic determination of 12 dyes in meat products by HPLC-UV-DIODE array detection

**DOI:** 10.1016/j.mex.2019.04.018

**Published:** 2019-04-22

**Authors:** Marco Iammarino, Annalisa Mentana, Diego Centonze, Carmen Palermo, Michele Mangiacotti, Antonio Eugenio Chiaravalle

**Affiliations:** a National Reference Center for the Detection of Radioactivity in Feed and Foodstuff, Istituto Zooprofilattico Sperimentale della Puglia e della Basilicata, Via Manfredonia 20, 71121, Foggia, Italy; b Department of the Sciences of Agriculture, Food and Environment, University of Foggia, Via Napoli, 25, 71122, Foggia, Italy

**Keywords:** 12 dyes in meat by HPLC-UV-DAD, Food safety, Colourings, Liquid chromatography

## Abstract

The use of food dyes in meat is regulated by the current European and non-European legislation, due to several food safety concerns. A reliable method for the quali-quantitative determination of 12 food dyes (Amaranth, Ponceau 4R, Carmine, Ponceau SX, Ponceau 3R, Allura Red AC, Carmoisine, Erythrosine, Sudan I, Sudan II, Sudan III and Sudan IV) in meat products, by high performance liquid chromatography coupled to UV diode array detection is presented. The extraction was accomplished by using acetonitrile, methanol, water, and ammonia, 50:40:9:1 (v/v/v/v) as the solvent and ultrasonic bath. The chromatographic separation was obtained with a C_18_ RP column eluted by a gradient of acetate buffer/acetonitrile. Good analytical performances characterized this method (Table 1), in terms of selectivity, sensitivity, accuracy and ruggedness. Both method precision (CV% range: 6%–15%) and recovery percentages (range: 86%–105%) resulted in compliance with Decision 2002/657/EC, and the expanded measurement uncertainties, estimated by a bottom-up approach, were in the range 6%–20%. All these results demonstrated that the procedure can be applied successfully for confirmation analyses of commercial meat products.

• 12 food dyes were determined in meat by new HPLC/UV-DAD method.
• The analytical method was fully validated for accurate confirmation analyses.
• Method accuracy, sensitivity, selectivity and ruggedness resulted satisfactory.

## Graphical abstract

**Figure:**
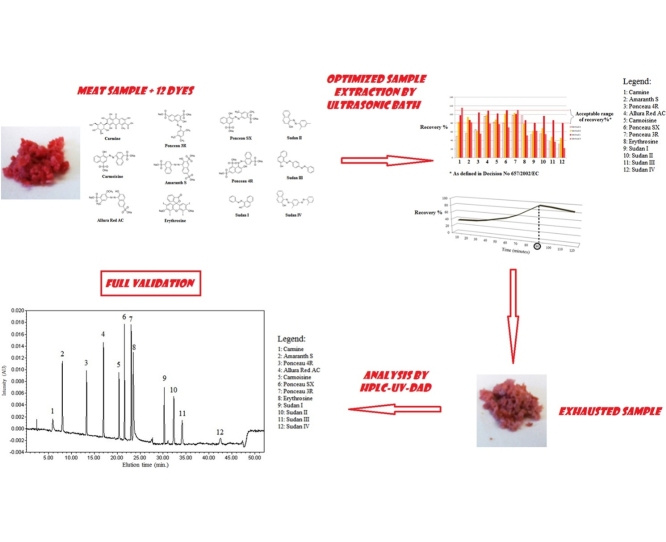

**Specifications Table**

**Table:** 

Subject area:	*Chemistry*
More specific subject area:	*Food Chemistry*
Method name:	*12 dyes in meat by HPLC-UV-DAD*
Name and reference of original method:	*M. Iammarino, A. Mentana, D. Centonze, C. Palermo, M. Mangiacotti, A.E. Chiaravalle, Simultaneous determination of twelve dyes in meat products: Development and validation of an analytical method based on HPLC-UV-diode array detection. Food Chem. 285 (2019) 1–9.*
Resource availability:	Amaranth, Ponceau 4R, Carmine, Ponceau SX, Ponceau 3R, Allura Red AC, Carmoisine, Erythrosine extra bluish, Sudan I, Sudan II, Sudan III, Sudan IV, sodium acetate anhydrous, acetic acid glacial, acetonitrile of HPLC grade, ammonium hydroxide (28–30%), methanol anhydrous, ultrapure water with a specific resistance of 18.2 MΩ-cm. HPLC system equipped with a PDA Detector, a micro vacuum degasser, an autosampler a column compartment and a C_18_ RP column (5 μm, 150 × 4.6 mm). Ultrasonic bath, vortex mixer.

## Dyes use in meat products

The topic “dyes in meat” is complex. This is due to both analytical lacks and toxicological evaluations still in progress. Indeed, some colourings may exhibit adverse reactions on humans. For instance, the Ponceau 3R, Amaranth and Scarlet GN were banned since toxic effects on rats were proved. Similarly, the Ponceau 4R was banned in the US once studies in attention deficit hyperactivity disorder (ADHD)-susceptible children were available [1]. Also Carmine, a widely-used dye in meat products, may cause allergy [2]. Given the lack of a comprehensive analytical method able to identify/quantify the most important dyes (both admitted and not admitted) in meat products, this work was focused on the development of a new procedure useful to fill this analytical gap.

### Materials and equipment

The analytical standards of 12 dyes were supplied by Sigma-Aldrich (Stenheim, Germany). The other reagents used were: sodium acetate anhydrous, acetic acid glacial, acetonitrile of HPLC grade, ammonium hydroxide (28–30%), methanol anhydrous. The solutions were prepared in ultrapure water with a specific resistance of 18.2 MΩ-cm, produced by a Milli-Q RG unit, Millipore (Bedford, MA, USA). The chromatographic determination of 12 dyes was accomplished by using a Waters™2690 Separations Module (Milford, US), equipped with a Waters™996 PDA Detector (Milford, US), a micro vacuum degasser, an autosampler and a column compartment. The C18 RP-Gold™column (5 μm, 150 × 4.6 mm, ThermoFisher, Waltham, USA), equipped with a drop-in guard cartridge (3 μm, 10 × 4 mm, ThermoFisher, Waltham, USA), was used as analytical column. The Waters®Millennium®32 software (Milford, MA) was used for data acquisition and elaboration. The complete extraction of dyes from the matrices (fresh meat and meat products) was obtained by using both an ultrasonic bath (HD 2200, Bandelin Electronic, Berlin) and a vortex shaker (VWR Digital Vortex Mixer, model 945312, VWR International, Radnor, Pennsylvania, USA) [1].

## Method details

A proper sample preparation was obtained both optimizing the composition of the extraction mixture and identifying the more efficient technique of extraction. The extraction mixture was formulated taking into account the solubility of dyes (azo dyes, Carmine and Sudan dyes soluble in methanol, water/ammonia and acetonitrile, respectively). 4 procedures of sample extraction (vortex shaker (A), magnetic stirrer (B), ultrasonic bath (C) and bain-marie (D)) were then compared.

The complete extraction of dyes from the matrices (fresh meat and meat products) was obtained by using the following procedure: the homogenized sample (2 g) was extracted with 20 mL of acetonitrile, methanol, water, ammonia 50:40:9:1 (v/v/v/v) in a 50 mL polypropylene tube, by using a vortex shaker for 2 min at 2500 rpm. The sample was then transferred in a 100 mL flask and placed in ultrasonic bath for 90 min (frequency: 100 Hz, T = 40 °C ± 4 °C). Lastly, the sample was re-placed in a 50 mL polypropylene tube and re-extracted by vortex shaker for 1 min at 2500 rpm. About 1.5 mL of extract were then microfiltered through 0.2 μm Minisart® NML surfactant-free cellulose acetate syringe filters and injected.

**Fig. 1:**
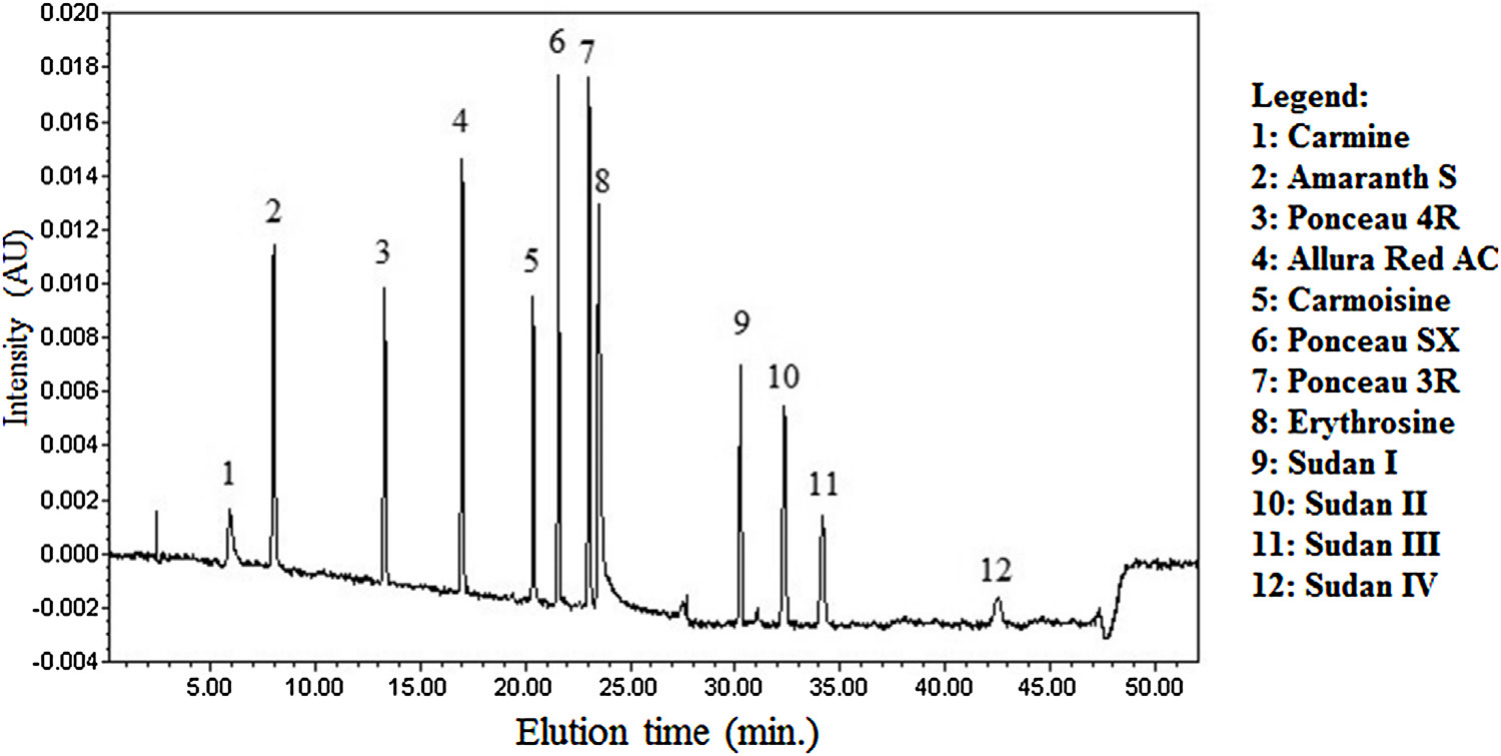
Example of chromatographic separation of 12 dyes (concentration: 5.0 mg L^−1^).

**Fig. 2:**
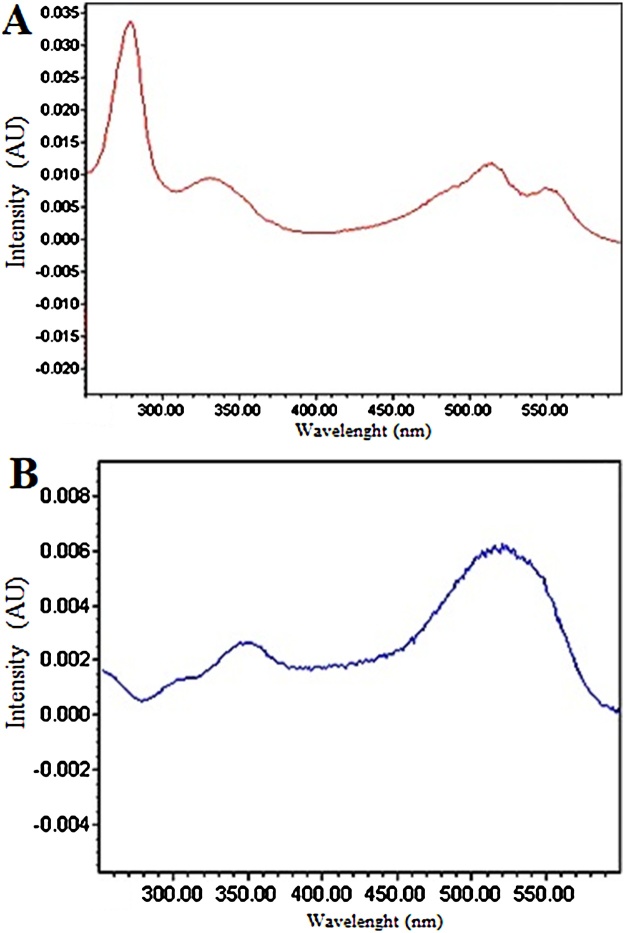
Absorbance spectrum examples in the range 250–600 nm: Carmine 80 mg kg^−1^ (A); Sudan IV 20 mg kg^−1^ (B).

Chromatographic conditions: The optimized chromatographic separation of 12 compounds (Fig. 1) was obtained by using a gradient of 0.02 M acetate buffer pH 7.0 (mobile phase A) and acetonitrile (mobile phase B). A flow rate of 1.2 mL min^−1^ and an injection volume of 10 μL were used. The elution gradient, with a total run time of 52 min, was the following: from 0% B to 15% B in 15 min, a subsequent gradient up to 34% B in 10 min, then up to 80% B in 1 min, isocratic for 21 min, gradient to 0% B in 1 min and a final 4-minute re-equilibration step at this mobile phase composition. The absorbance signal was detected at 520 nm. In Fig. 1, the chromatogram of a standard solution of 12 dyes is shown. The retention times repeatability, expressed as CV% (n = 6), was in the range 0.5 (Sudan II) – 3.3 (Allura Red AC). Setting the acquisition wavelength range from 250 to 600 nm, the diode array detector allowed the collection of the absorbance spectrum of each food dye. In Fig. 2, two examples of absorbance spectra are shown.

### Method validation

This analytical method was validated following the Thompson harmonized validation guidelines [3], in agreement with Regulation 2017/625/EU and Decision 2002/657/EC. The following analytical parameters were evaluated: linearity, selectivity, detection and quantification limits (LODs and LOQs), accuracy, ruggedness and measurement uncertainty.

Method linearity was verified, in terms of determination coefficient (r^2^>0.99), by injecting five standard solutions at the following concentrations: 1.0, 2.5, 5.0, 10.0 and 20.0 mg L^−1^ for Amaranth, Ponceau 4R, Carmoisine, Ponceau SX, Erythrosine and Sudan IV; 0.5, 1.0, 2.5, 5.0 and 10.0 mg L^−1^ for Allura Red AC, Ponceau 3R, Sudan I, Sudan II and Sudan III; 5, 10, 20, 40, 80 mg L^−1^ for Carmine.

The calibration curves were also elaborated for estimating the LOD and LOQ values. The following equations were used: LOD = 3.3s_a_/b and LOQ = 10s_a_/b, where s_a_ is the standard deviation of the intercept and b is the slope of the linear regression.

Method selectivity was verified by analyzing 20 samples of fresh meat and meat products. The absence of interfering peaks in the retention time-window of interest (±2.5% of each dye retention time) was ascertained, confirming method selectivity.

**Fig. 3:**
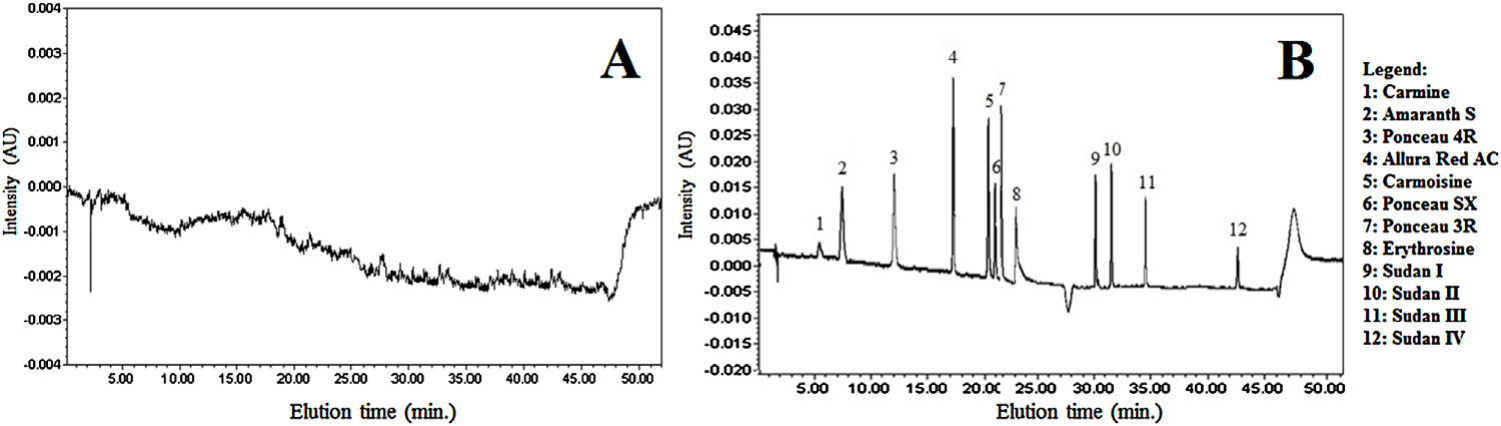
Chromatograms examples: Pork fresh meat sample (A); Pork fresh meat sample spiked with 100 mg kg^−1^ of 12 dyes (B).

Method accuracy was assessed as precision (CV%) and trueness (recovery%) by analyzing three sets of blank pork fresh meat samples (six replicates each), fortified with each dye at three levels: 25, 50 and 100 mg kg^−1^. In Fig. 3 a comparison between pork fresh meat samples fortified and not fortified with 12 dyes is shown.

Method ruggedness was evaluated in terms of application field (matrix to analyze). Twelve independent new experiments were carried out by analyzing cow fresh meat, salami and seasoned sausage samples (four each) and then comparing the obtained results with those resulting from the validation matrix (pork fresh meat). These types of meat were chosen taking into account the meat products in which the addition of some food dyes is admitted. These samples were fortified with 25 mg kg^−1^ of each dye (except Carmine and Sudan IV, fortified at 100 and 50 mg kg^-1^, respectively). This approach, proposed by Youden & Steiner [4], is based on the standard deviation of difference comparison among different matrices. If this parameter is comparable (2 tails F test, at 7 and 11 degrees of freedom, 95% confidence level) with the method precision estimated for the validation matrix, this variation has no effect on the analytical performances and the method application field may be extended accordingly.

The measurement uncertainty was calculated by using the “bottom-up” approach, taking into account the uncertainty estimated for each step of the analytical procedure.

All validation parameters well comply with European Legislation. The ruggedness studies confirmed that method is applicable to pork and beef fresh meat, salami and seasoned sausage. In Table 1, the performances and the validation parameters that characterize this method are reported. An in-depth description of the validation procedure was published elsewhere [1].

**Table 1:** Method performances and validation parameters.

Dye	Determination coefficient (r^2^)	LOQ (mg kg^−1^ in matrix)	Mean Recovery % (n = 18)^a^	Mean CV% (n = 18)^a^	Expanded measurement uncertainty (k = 2)
***Carmine***	0.999	13	101	6	6%
***Amaranth***	0.999	18	103	12	13%
***Ponceau 4R***	0.999	15	95	14	14%
***Allura Red AC***	0.998	11	93	15	12%
***Carmoisine***	0.998	22	100	11	13%
***Ponceau SX***	0.998	22	105	7	18%
***Ponceau 3R***	0.997	12	99	10	14%
***Erythrosine***	0.998	23	89	11	17%
***Sudan I***	0.995	16	92	11	14%
***Sudan II***	0.992	22	91	9	20%
***Sudan III***	0.999	4	86	13	10%
***Sudan IV***	0.999	19	90	12	10%

a Three fortification levels (6 repetitions each).

## Supplementary material and/or Additional information

None.

## References

[1] Iammarino M., Mentana A., Centonze D., Palermo C., Mangiacotti M., Chiaravalle A.E. (2019). Simultaneous determination of twelve dyes in meat products: development and validation of an analytical method based on HPLC-UV-diode array detection. Food Chem..

[2] Takeo N., Nakamura M., Nakayama S., Okamoto O., Sugimoto N., Sugiura S., Sato N., Harada S., Yamaguchi M., Mitsui N., Kubota Y., Suzuki K., Terada M., Nagai A., Sowa-Osako J., Hatano Y., Akiyama H., Yagami A., Fujiwara S., Matsunaga K. (2018). Cochineal dye-induced immediate allergy: review of Japanese cases and proposed new diagnostic chart. Allergol. Int..

[3] Thompson M., Ellison S.R.L., Wood R. (2002). Harmonized guidelines for single laboratory validation of methods of analysis. Pure Appl. Chem..

[4] Youden W.J., Steiner E.H. (1975). Statistical Manual of the AOAC.

